# Use of Advanced Artificial Intelligence in Forensic Medicine, Forensic Anthropology and Clinical Anatomy

**DOI:** 10.3390/healthcare9111545

**Published:** 2021-11-12

**Authors:** Andrej Thurzo, Helena Svobodová Kosnáčová, Veronika Kurilová, Silvester Kosmeľ, Radoslav Beňuš, Norbert Moravanský, Peter Kováč, Kristína Mikuš Kuracinová, Michal Palkovič, Ivan Varga

**Affiliations:** 1 Department of Stomatology and Maxillofacial Surgery, Faculty of Medicine, Comenius University in Bratislava, 81250 Bratislava, Slovakia; 2 Department of Simulation and Virtual Medical Education, Faculty of Medicine, Comenius University, Sasinkova 4, 81272 Bratislava, Slovakia; helena.svobodova@fmed.uniba.sk; 3 forensic.sk Institute of Forensic Medical Analyses Ltd., Boženy Němcovej 8, 81104 Bratislava, Slovakia; benus1@uniba.sk (R.B.); norbert.moravansky@forensic.help (N.M.); pkovac@gmail.com (P.K.); 4 Department of Genetics, Cancer Research Institute, Biomedical Research Center, Slovak Academy Sciences, Dúbravská Cesta 9, 84505 Bratislava, Slovakia; 5 Faculty of Electrical Engineering and Information Technology, Slovak University of Technology, Ilkovičova 3, 81219 Bratislava, Slovakia; veronika.hanuskova@gmail.com; 6 Deep Learning Engineering Department at Cognexa, Faculty of Informatics and Information Technologies, Slovak University of Technology, Ilkovičova 2, 84216 Bratislava, Slovakia; xkosmel@stuba.sk; 7 Department of Anthropology, Faculty of Natural Sciences, Comenius University in Bratislava, Mlynská dolina Ilkovičova 6, 84215 Bratislava, Slovakia; 8 Institute of Forensic Medicine, Faculty of Medicine, Comenius University in Bratislava, Sasinkova 4, 81108 Bratislava, Slovakia; 9 Department of Criminal Law and Criminology, Faculty of Law Trnava University, Kollárova 10, 91701 Trnava, Slovakia; 10 Institute of Pathological Anatomy, Faculty of Medicine, Comenius University in Bratislava, Sasinkova 4, 81108 Bratislava, Slovakia; kristina.kuracinova@fmed.uniba.sk (K.M.K.); padidivecenter@gmail.com (M.P.); 11 Forensic Medicine and Pathological Anatomy Department, Health Care Surveillance Authority (HCSA), Sasinkova 4, 81108 Bratislava, Slovakia; 12 Institute of Histology and Embryology, Faculty of Medicine, Comenius University in Bratislava, 81372 Bratislava, Slovakia; ivan.varga@fmed.uniba.sk

**Keywords:** forensic medicine, forensic dentistry, forensic anthropology, 3D CNN, AI, deep learning, biological age determination, sex determination, 3D cephalometric, AI face estimation, growth prediction

## Abstract

Three-dimensional convolutional neural networks (3D CNN) of artificial intelligence (AI) are potent in image processing and recognition using deep learning to perform generative and descriptive tasks. Compared to its predecessor, the advantage of CNN is that it automatically detects the important features without any human supervision. 3D CNN is used to extract features in three dimensions where input is a 3D volume or a sequence of 2D pictures, e.g., slices in a cone-beam computer tomography scan (CBCT). The main aim was to bridge interdisciplinary cooperation between forensic medical experts and deep learning engineers, emphasizing activating clinical forensic experts in the field with possibly basic knowledge of advanced artificial intelligence techniques with interest in its implementation in their efforts to advance forensic research further. This paper introduces a novel workflow of 3D CNN analysis of full-head CBCT scans. Authors explore the current and design customized 3D CNN application methods for particular forensic research in five perspectives: (1) sex determination, (2) biological age estimation, (3) 3D cephalometric landmark annotation, (4) growth vectors prediction, (5) facial soft-tissue estimation from the skull and vice versa. In conclusion, 3D CNN application can be a watershed moment in forensic medicine, leading to unprecedented improvement of forensic analysis workflows based on 3D neural networks.

## 1. Introduction

Conventional forensic analysis is based on forensic expert’s manual extraction of information. Forensic expert provides opinions established on medical and other fields of current knowledge combined with personal work experience. This is not only time-consuming, albeit frequently affected by subjective factors that are tough to overcome [1].

The main purpose of this paper is to analyze and introduce a very promising line of research applicable to forensic anthropology and various traditional sectors of forensic medicine. The application of artificial intelligence (AI) is a new trend in forensic medicine and a possible watershed moment for the whole forensic field [1,2,3,4,5,6].

This chapter paper explains basic terminology, principles and the current horizon of knowledge. The methodology chapter presents the novel clinical workflow based on implementing three-dimensional convolutional neural network (3D CNN) algorithms [7,8,9]. The input is full head cone-beam computer tomography scans (CBCT) in the Digital Imaging and Communications in Medicine format (DICOM) [9,10,11,12,13,14]. The methodology chapter describes technical data preparation for 3D CNN utilization in the following practical aspects from forensic medicine:

1. Biological age determination [7,8,15,16,17,18,19,20,21,22,23,24,25,26,27,28,29,30,31]
2. Sex determination [32,33,34,35,36,37,38,39,40]
3. Automatized 3D cephalometric landmark annotation [41,42,43,44,45,46,47,48,49,50,51,52,53,54,55,56,57,58]
4. Soft-tissue face prediction from skull and in reverse [59,60,61,62,63,64,65,66,67,68,69,70,71,72,73,74,75,76,77]
5. Facial growth vectors prediction [13,59,78,79,80,81,82,83,84,85,86,87,88,89,90]

The result of this paper is a detailed guide for forensic scientists to implement features of 3D CNN to forensic research and analyses of their own (in five themes described above). This resulting practical concept—possible workflow shall be useful for any forensic expert interested in implementing this advanced artificial intelligence feature. This study is based on the worldwide review of 3D CNN use-cases that apply to clinical aspects of forensic medicine

This article’s secondary objective is to inspire forensic experts and approximate them to implement three-dimensional convolutional neural networks (3D CNN) in their forensic research in the fields of age, sex, face and growth determination.

### 1.1. Basic Terminology and Principles in Era of AI Enhanced Forensic Medicine

Artificial intelligence has brought new vigor to forensic medicine, but at the same time also some challenges. AI and forensic medicine are developing collaboratively and advanced AI implementation until now required extensive interdisciplinary cooperation. In the era of big data [3], forensic experts shall become familiar with these advanced algorithms and understand used technical terms.

For many forensic experts, the current benefits of advanced AI processes are still unknown. For example, automated AI algorithms for skull damage detection from CT [91] or soft-tissue prediction of a face from the skull [66,67,89,92] are still a mystery to many outstanding forensic scientists. Enabling them would catapult forensic research to a new era [1].

A Convolutional Neural Network (CNN) is a Deep Learning algorithm that can take in an input image, assign importance (learnable weights and biases) to various aspects/objects in the image, and differentiate one from the other.

**Figure 1:**
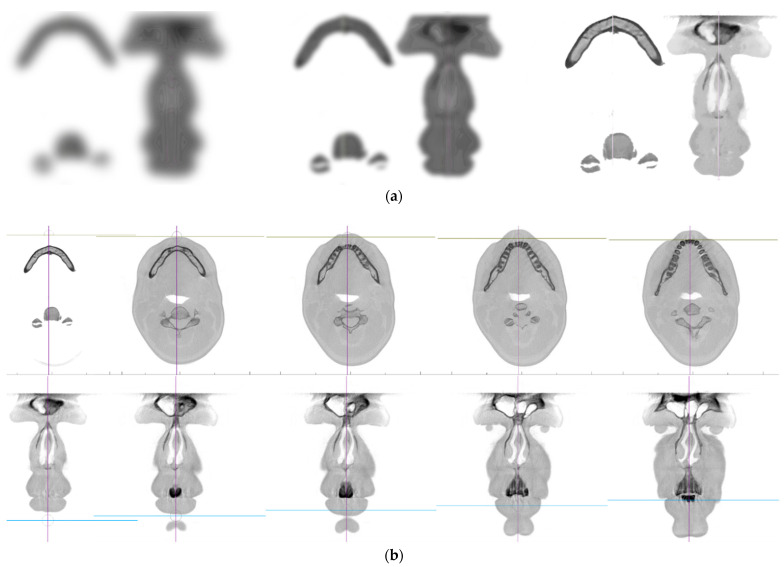
(**a**) Recognition of objects. Try, using your imagination, to recognize the objects on the three blurred variants of the same anatomical slice. Convolutional Neural Networks (CNNs) work similar to our visual brain when trying to recognize these objects. (**b**) Our recognition of objects on the picture is significantly improved when more layers—slices are added thus providing further context with the 3rd dimension. In the top row is recognizable intersection of the mandible and vertebra and on the lower row is recognizable slice of the face. 3D CNN recognition is similarly improved with providing context of depth.

CNN is an efficient recognition algorithm that is widely used in pattern recognition and image processing. It has many features such as simple structure, less training parameters and adaptability. CNN is a supervised type of Deep learning, most preferable used in image recognition and computer vision (Figure 1a,b).

Compared to its predecessors, the main advantage of CNN is that it automatically detects the crucial features without any human supervision. For example, given many pictures of cats and dogs, it learns distinctive features for each class. CNN is also computationally efficient.

3D CNN is used to extract features in 3 Dimensions or establish a relationship between 3 dimensions. A 3D CNN is simply the 3D equivalent: it takes as input a 3D volume or a sequence of 2D frames (e.g., CBCT scan).

In terms of Neural Networks and Deep Learning: Convolutions are filters (matrix/vectors) with learnable parameters used to extract low-dimensional features from input data. They have the property to preserve the spatial or positional relationships between input data points.

**Figure 2:**
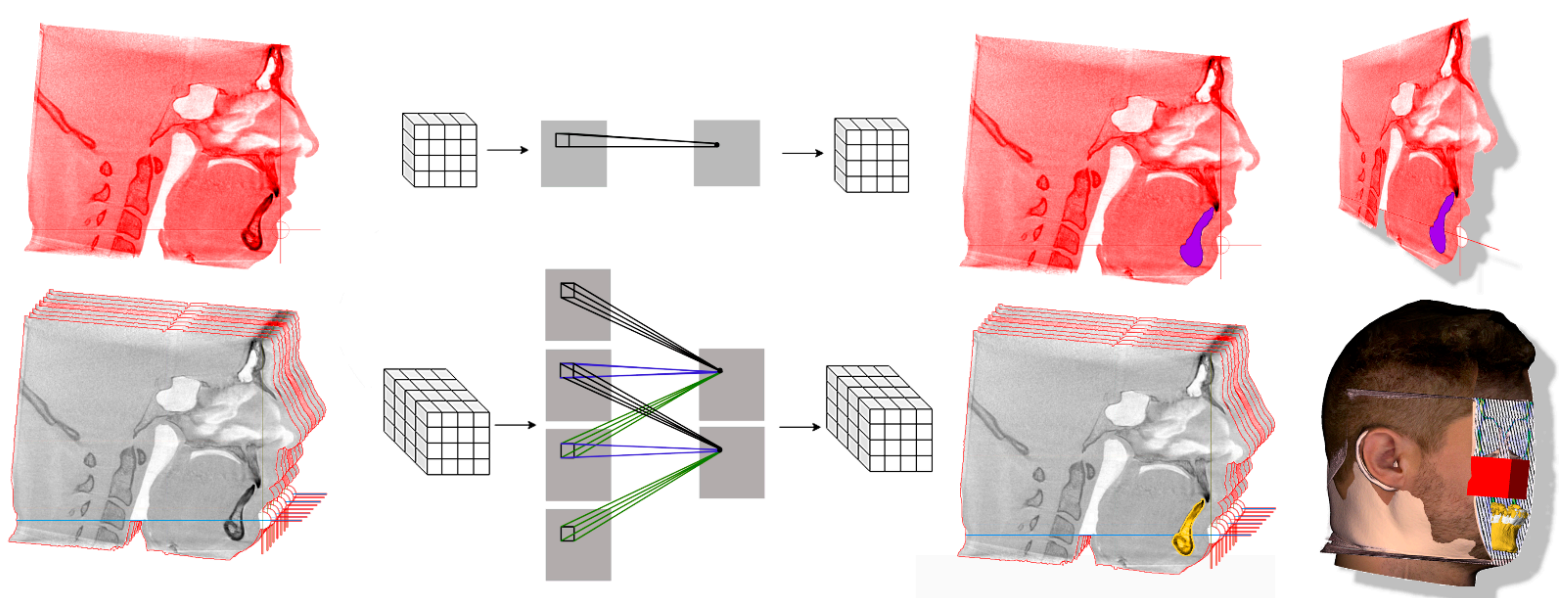
The comparison of 2D CNN (**above**) and 3D CNN (**below**). 3D CNN works with 3rd dimension and can reconstruct shapes from the CBCT 2D slides. The sequence of 2D pictures where the 3rd dimension is time, we speak of a common video sequence that can be a subject of 3D CNN analysis too.

2D CNNs predict segmentation maps for DICOM slices in a single anatomical plane. 3D CNNs address this issue by using 3D convolutional kernels to make segmentation predictions for a volumetric patch of a scan (Figure 2).

In 3D convolution, a 3D filter can move in all 3-directions (height, width, channel of the image). At each position, the element-wise multiplication and addition provide one number. Since the filter slides through a 3D space, the output numbers are also arranged in a 3D space. The output is then 3D data.

**Figure 3:**
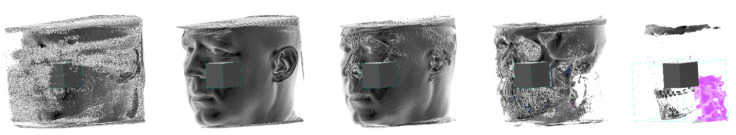
The example of the process of defining ranges for particular visualized tissues is called “thresholding”.

**Figure 4:**
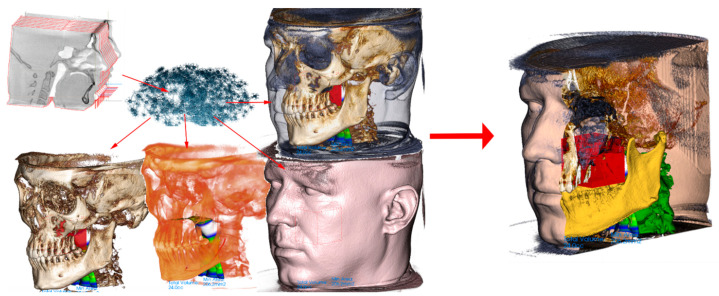
The examples of the segmentation process on the CBCT data based on defining ranges for particular tissues thus defining 3D structures such as airway, nerve canal, face surface or bone structures.

The recognition of similar structures from the CBCT is based on their similar opacity on the X-ray classified by the Hounsfield scale. The process of defining ranges for particular tissues is called “thresholding”, which is prior to final the segmentation (Figure 3). Setting different thresholds for segmentation preprocessing step allows segmentation of different structures such as soft tissues (skin, airway, sinuses), nerves (inferior alveolar nerve, dental pulp), bones (mandible, maxilla or cervical vertebras) and many other (Figure 4).

**Figure 5:**
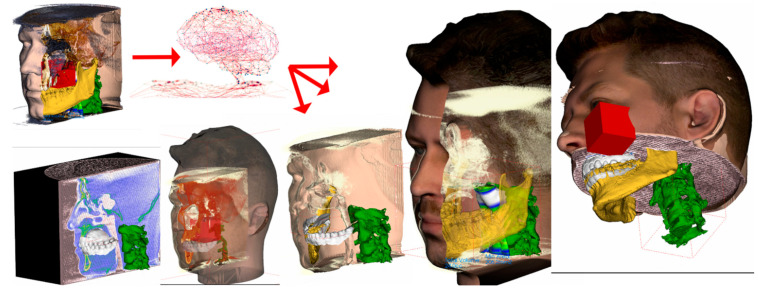
The example of 3D data augmentation in a sense of mapping another 3D scans on the segmented structures. Facial 3D scan with texture mapped on the segmented face surface from CBCT and intraoral scan of teeth and gums mapped on tooth surfaces from the CBCT. Finally merged into complex set of 3D models. Training of 3D CNN with such a complex 3D virtualized model has never been performed before and is worth a consideration.

The segmentation of original CBCT data can result in the definition of various 3D structures involved in 3D CNN training, or these 3D structures can serve as anchors for mapping another 3D scan, such as an intraoral optical scan or extraoral scan that includes texture. All these three data sources can be merged, and the 3D CNN network can work with unprecedented data that include wider face regions from face scan or morphological information on teeth and gums (Figure 5).

### 1.2. Overview of Used Artificial Intelligence for Forensic Age and Sex Determination

Traditional forensic analyses of age, gender and facial appearance are based on forensic expert manually acquiring information that provides the identification established on expert`s medical and biological knowledge and mathematical calculations [93,94,95]. In forensic outputs, the experiences of the investigator subjectivity and fatigue and emotions play a role [93,94,95]. To have forensic expert well trained on thousands of skulls of all possible ethnicities, would take a lifetime. Possible bias sourcing from fatigue, limited training dataset, emotional engagement or human calculation error cannot be absolutely eradicated with human forensic expert. Implementation of artificial intelligence (AI) can limit all these mentioned sources of possible bias. Machine learning works based on models that mimic neurons in the brain and can learn from experiences and solve complex problems. It is not influenced by subjective judgment; it does not become tired and does not use emotions and thus can work more efficiently [96,97,98].

AI usage is not without risks of undesired side effects. AI may become biased in the same way as a human forensic expert, depending on the source data used for AI training [99]. Obermeyer at el. found evidence of bias in a healthcare algorithm responsible for 200 million people, which systemically prevented almost 30% of eligible black patients from receiving additional care by giving lower risk scores to black patients than white patients with equal diagnoses [100].

Many studies in forensic science have been conducted in recent years, and some recent studies are beginning to focus on neural networks [101]. These studies were mainly aimed at determining the age and sex of postmortem skeletal remains and living people. Age and gender assessment active, used to identify victims, determine criminal liability or identify persons without legal documentation [8,102]. There is considerable interest in accelerating identification procedures, and experts are involved in machine learning in forensic procedures. They use X-ray images [103,104,105,106,107,108], MRI images [8,109], photography [90,110,111], CT scans [112,113,114,115,116,117] of the head or other bones such as the collarbone, femur, teeth, etc. and use databases to teach artificial intelligence to identify people’s age or gender. Pham et al. [113] examined age using the femur and mandible for neuronal networks. The femur could play a key role in predicting adulthood, especially the shape of the femoral head and bone densitometry. They used 814 whole-body post-mortem computed tomography (CT) scans to obtain results: 619 men, 195 women aged 20 to 70 years. They omitted subjects with fractures. Each CT output was in digital imaging and communication in medicine (DICOM) format [11,12]. The extracted femur and mandible data were preprocessed to create a 3D voxel inserted into a neural network model. Using this approach, the mean absolute error (MAE) of the mandible age identification was 7.07 years, and the MAE calculated from a femur age determination was 5.74 years. The combination of both approaches reached an excellent result—MAE = 5.15 years. CT scans were also used for learning and age determination in a study by Farhadian et al. [115]. AI determined the age learned from CT scans of 300 subjects aged 14 to 60 years of the canine teeth. In this study, they compared the methodology of neural networks with a regression model. The MAE for neural networks was 4.12 years, and the MAE for the regression model was 8.17 years, which demonstrated the higher accuracy of neural networks. Mauer et al. [102] aimed to develop a fully automated and computerized method for age estimation based on the knee’s 3D magnetic resonance imaging (MRI). They had 185 coronal and 404 sagittal MR images of Caucasian men aged 13 to 21 years. The best result obtained was a MAE of 0.67 ± 0.49 years and an accuracy of 90.9%. Here it can be seen that the group with a minor age variance more accurately determines the age of the individuals. A similar study was performed by Stern et al. performed a similar study [109] where 328 MR images were used for learning neural networks and subsequent age detection. Age was reported with a MAE of 0.37 ± 0.51 years for the age range of individuals ≤ 18 years.

Several research teams have tried neural network learning based on X-ray images [103,104,108]. Guo et al. [103] used 10,257 samples of dental orthopantomograms and, similar to Farhadian et al. [115], compared logistic regression linear models for each legal age limit (14, 16 and 18 years) with the neural network. The results showed that neural networks work better (linear regression models: 92.5%, 91.3% and 91.8% and neural networks: 95.9%, 95.4% and 92.3% success rate for age limits 14, 16 and 18 years). In Stepanovsky et al. [105] used 976 orthopantomography (662 men, 314 women) of people aged 2.7 to 20.5 years to learn neural networks. The results were very favorable, and the average absolute error (MAE) was below 0.7 years for both men and women. Vila-Blanco et al. [106] used landmarks on the mandible to search for patterns by neural networks. The age estimate reached an accuracy of 87.8%, and the MAE was only 1.57 years. De Tobel et al. [107] used panoramic molar panoramic radiographs to estimate age. The accuracy of the results was, on average, MAE = 0.5. Boedi et al. [108] later conducted a similar study with similar results. Li et al. [104] used 1875 X-ray images of the pelvis as a basis for evaluating bone age through deep learning. The age of the people whose X-rays were used to teach the model was 10 to 25 years. The performance of the model was MAE = 0.94 years.

More studies modelled gender determination using AI. Bewes et al. [42] used neural networks for this purpose with a detection accuracy of 95%. However, they trained them on 900 skull scans from CT scans. Oner et al. [114] achieved the same goal by using CT images of the sternum transmitted to the orthogonal plane for learning neural networks. They used 422 thin sections of thoracic CT scans (213 females, 209 males) with an age range of 27–60 years. The accuracy of gender prediction was 0.906, and the confidence interval of 94%. The success rate was higher than that achieved by linear models. Etli et al. [116] compared several methods in the study. They used CT scans with sacral and coccyx metric parameters of 480 patients. They used one-dimensional discriminant analysis, linear discriminant functional analysis, sequential analysis of discriminant function and multilayer perceptron neural networks. The maximum accuracy for each method was 67.1% for one-dimensional discriminant analysis, 82.5% for linear analysis of the discriminant function, 78.8% for sequential analysis of the discriminant function, and 86.3% for multilayer perceptron neural networks.

Gender classification was also discussed by Liew et al. [111]. The maximum accuracy for each method was 67.1% for one-dimensional discriminant analysis, 82.5% for linear analysis of the discriminant function, 78.8% for sequential analysis of the discriminant function, and 86.3% for multilayer perceptron neural networks. Gender classification was also discussed by Liew et al. [111]. They used grayscale images of 200 men and 200 women for analysis. The classification performance reached 98.75% and 99.38% in the facial databases SUMS and AT&T. To estimate the sex of infants in the study of Ortega et al. [110] used 2D photographs of the ilium of 135 individuals aged 5 months to 6 years were used. The accuracy was 59% compared to 61% for the specialist. In addition, Porto et al. [88] sought to determine the legal age of offenders at 14 and 18 years as Guo et al. [103]. They based on a database of photographs of 18,000 faces of men and women based on photo anthropometric indices from cephalometric landmarks marked and checked by forensic experts. The accuracy of age determination by neural networks was 0.72 with an age interval of 5 years and for the estimation of the age group higher than 0.93 and 0.83 for the threshold values of 14 and 18 years.

It is almost unbelievable how accurately neural networks can determine age or gender compared to commonly used methods. Therefore, we emphasize their use in forensic practice [9,46,50,117].

Regarding the Skeletal age estimation for forensic purposes, we consider ourselves useful for the direction of the 3D CNN on particular areas of the head and neck. Various experts published research on age estimation by measuring open apices in teeth, stage of teeth eruption, frequently of third molars or canine tooth/pulp ratio [6,17,18,20,21,23,24,25,27,29,31]. In general, teeth are frequently used for age assessment, but they are not the only structures in the skull to be considered. It is known that the shape of the frontal sinus can be an essential tool in personal forensic identification and is linked together with the cranial base to growth changes that can be evaluated [6,118]. Another typical location for skeletal age assessment in the head and neck X-ray diagnostics region is the stage of cervical vertebrae maturation [23,119]. Deep learning has been already implemented in this area [83]. Extensive research is published regarding skeletal age expert estimation Pinchi et al. [120,121,122,123,124,125,126] mainly combines dental and skeletal findings. If the 3D CNN fails to identify these valuable areas, we still have the opportunity to direct the focus on these areas.

Regarding forensic medico-legal aspects, the perspective on natural development estimated by AI algorithms is always relevant, especially in the situation of trauma or other damage that conflicted with this estimated development. AI is now used to evaluate CT scans of lungs and to predict the deterioration of COVID-19 patients in the emergency department [127,128,129].

In this case, 3D CNN algorithms can automatically evaluate not only hard-tissue structures and search for inapparent damage that could have been responsible for a sudden death incident [91,130].

### 1.3. Artificial Intelligence Implementation in 3D Cephalometric Landmark Identification

**Figure 6:**
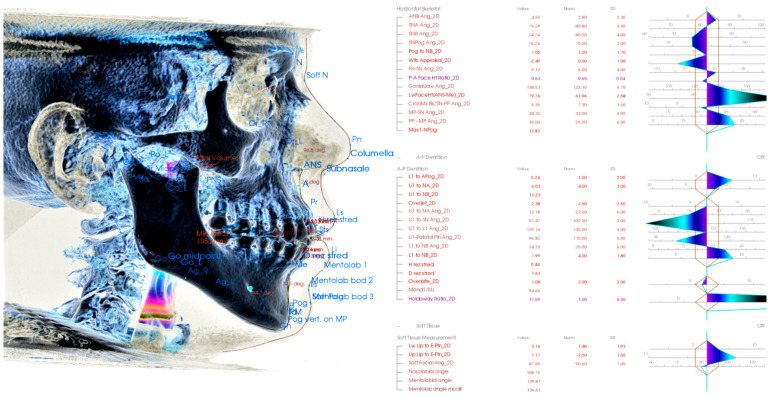
Example of 3D cephalometric analysis where orthodontist identifies more than 50 points and the hard- and soft-tissues analyzed. Humans chose these points as the most reproducible on X-ray. These might not be ideal representatives of head and neck structures linked with biological ageing or sexual dimorphism.

Analysis of complex cranial and facial structures is a domain of orthodontics. Historically they are fundamental for proper treatment planning, and they represent lines, angles, planes on the axilla-facial structures identifiable, especially on the X-ray (typically lateral X-ray). There is massive research regarding cephalometric parameters and their values. Observer defines the points, and their interobserver error are the main weakness of cephalometric analysis (Figure 6). Anthropometry in Forensic Medicine and Forensic Science is frequently used for sex and biological age determination and other purposes [129,130,131].

As the various cephalometric parameters (angles, ratios and distances) were well researched, and some are proven to be related to age, sex or growth, they are a frequent springboard for many research studies focused on facial parameters. Implementation of AI in cephalometric analysis has been published [132,133,134,135,136]. The question is whether the 3D CNN trained networks will find even better regions and soft- and hard-tissue features on CBCTs when autonomously searching for links between voxel structures and the age or sex. Either way, the reliable automatized 3D cephalometric algorithm precisely identifying particular points with extreme repeatability would be a helpful tool not intended to replace humans in cephalometric points identifications. However, the human error is impossible to cancel completely as the interobserver error.

### 1.4. Artificial Intelligence Implementation in Soft-Tissue Face Prediction from Skull and Vice Versa

Reconstruction of the face from the skull is an age-old desire of forensic experts. Current methods of not implementing AI are very limited. Prediction of soft tissues according to the hard tissues of the skull and vice versa can be significantly improved upon big-data training of 3D CNN with supplementary metadata about age, sex, BMI or ethnicity. New algorithms to perform facial reconstruction from a given skull has forensic application in helping the identification of skeletal remains when additional information is unavailable [29,64,66,67,68,69,70,72,73,85,86,88,89,92,137]. Implementation of 3D CNN can also unintentionally open pandora box of guided improving the morphology of the facial soft-tissues. From a socio-psychological standpoint, this is regarded as an important therapeutic goal in modern orthodontic treatments. Currently, many of the algorithms implemented in commercially available software present ability to predict profile changes grounded on the incorrect assumption that the amount of movement of hard-tissue and soft-tissue has a proportional relationship [82].

**Figure 7:**
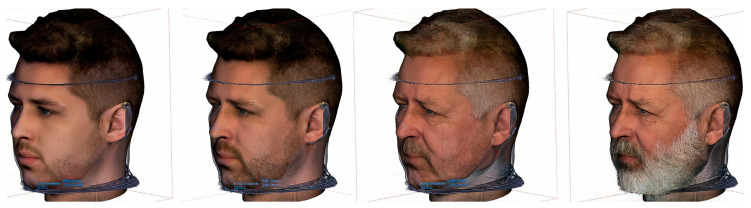
Example of CNN use of the FaceApp AI application to render the face mapped on CBCT to look younger or older. The algorithms changed just the texture and not the 3D mask, however this is probably only a matter of time. 2D face morphing based on AI or face swapping in popular videos are available and popular already a couple of years. Original face is the 2nd one.

The beauty industry has seen rapid growth in multiple countries, and due to its applications in entertainment, the analysis and assessment of facial attractiveness have received attention from scientists, physicians, and artists because of digital media, plastic surgery, and cosmetics. An analysis of techniques is used to assess facial beauty that considers facial ratios and facial qualities as elements to predict facial beauty [81,82,138,139,140]. A popular and famous free app using AI is FaceApp, which uses neural networks to enhance, age or otherwise change 2D digital photos of users uploading them using this application (Figure 7). Using the 3D CNN approach was not yet implemented despite iPhones having a 3D lidar scanner to acquire a 3D soft-tissue scan of the user’s face. From a forensic aspect, this era of digital 2D face manipulation brought deep-fake videos and images. Detecting manipulated facial images and videos is an increasingly important topic in digital media forensics [118,141]. Any face can be used in the fake video, or unlimited numbers of nearly authentic pictures, including fake social media profiles, can be created. AI is used in forensic evaluation for facial forgery detection and manipulated region localization [118].

This paper most complex AI application is the final 5th theme—“Facial growth vectors prediction”. The authors of this paper addressed it for various reasons. Firstly, it is fundamentally different from the first four themes. Secondly, it requires the most complex implementation of AI strategies. To our knowledge, this is only the second paper in the world that handles the problem of facial growth prediction with ML methods and absolutely the first paper to consider a 3D CNN for facial growth predictions.

Prediction of natural growth is compared to typically forensic topics such as human remains reconstruction and identification or age and sex determination rather less familiar topic. Mainly because despite numerous research attempts to predict facial growth, a satisfactory method has not been established yet, and the problem still poses a challenge for medical experts [142,143,144]. Predicting natural growth and later ageing is relevant for orthodontic therapy planning and from a forensic aspect. Any damage to the head and neck region that would affect otherwise natural growth or simple ageing could be evaluated. The effect of such a trauma could be in the future forensically quite accurately evaluated.

In 1971 Hirschfeld and Moyers published an article named “Prediction of craniofacial growth: the state of the art” [144]. This was one of the first attempts for facial growth predictions. The authors concluded that there are many reasons why they fail to predict craniofacial growth, and some they named persisted until today. They expressed doubts that we have not always measured the right thing. They also pointed out the lack of biological meaning for many traditional cephalometric measures. They have also pointed to the heritability of attained growth in the face and predicted the future importance of craniofacial genetics. The future that comes proved them correct in many aspects. Since these first attempts to predict the facial growth direction over half of a century ago, we did not become much better in facial growth prediction [142]. The complexity of the problem is challenging.

The only study that was focused on the prediction of the facial growth direction with Machine Learning methods and has been published so far is a paper with its pre-print [90,145] from 2021 by Stanislaw Kazmierczak et al. The outcomes of this paper are not impressive regarding facial growth prediction, albeit inspiring in the method of evaluation. The authors of this novel paper [94] performed feature selection and pointed out the attribute that plays a central role in facial growth. Then they performed data augmentation (DA) methods. This study is discussed in more detail later in this paper.

## 2. 3D Convolutional Neural Networks and Methods of Their Use in Forensic Medicine

### 2.1. Hardware and Software Used

CBCT scans analyzed for this paper were made on one machine: i-CAT™ FLX V17 with the Field of View (FOV) of 23 cm × 17 cm with technical parameters and settings Table 1.

Medical software used for DICOM data processing and analysis was Invivo™ 6 from Anatomage Inc., Silicon Valley, Thomas Road Suite 150, Santa Clara, CA 95054, USA.

Software for the AI solution base we have used the Python programming language along with 3 deep learning libraries—TensorFlow 2, PyTorch and MONAI. As for the hardware, the whole AI system is powered by multiple GPUs.

### 2.2. Main Tasks Definitions

Task 1—Age estimation from whole 3D CT scan image

Definition: the task is to estimate the approximate age of a person from a whole head 3D CBCT scan

Proposed method: build regression model represented by a 3D deep neural network that has the current state of the art network architecture as a backbone

Metrics: Mean Absolute Error (MAE) and Mean Squared Error (MSE) (see Section Evaluation)

Task 2—Sex classification from thresholded soft and hard tissues

Definition: the task is to classify input 3D CBCT scans (whole head or experimentally segmented parts) into one of 2 predefined categories—female and male

Proposed method: build classification model represented by 3D deep neural network based on convolutional layers and outputs class probabilities for both targets

Metrics: Accuracy and Confusion Matrix (CM) (other metrics such as precision, recall and F1 score will be evaluated in a later phase)

Task 3—Automatization of cephalometric measurements

Definition: the task is to create an automated system able to tag cephalometric landmarks on whole head 3D CT scan

Proposed method: build object detection model based on 3D neural network that estimates cephalometric measurements automatically

Metrics: Mean Absolute Error (MAE) and Mean Squared Error (MSE) (see Section Evaluation)

Task 4—Soft-tissue face prediction from skull and vice versa

Definition: the task is to create an automated system that predicts the distance of the face surface from the bone surface according to the estimated age and sex. 3D CNN to be trained on whole-head CBCTs of soft-tissue and hard-tissue pairs. *CBCTs with trauma and other unnatural deformations shall be excluded.

Proposed method: build a generative model based on Generative Adversarial Network that synthesizes both soft and hard tissues

Metrics: the slice-wise Frechet Inception Distance (see Section Evaluation)

Task 5—Facial growth prediction

Definition: the task is to create an automated system that predicts future morphological change in defined time for the face’s hard- and soft tissues. This shall be based on two CBCT input scans of the same individual in two different time points. The second CBCTs must not be deformed with therapy affecting morphology or unnatural event. This already defines the extremely challenging condition. There is a high possibility of insufficient datasets and the necessity of multicentric cooperation for successful training of 3D CNN on this task.

Proposed method: In this final complex task, the proposed method builds on previous tasks. We strongly recommend adding metadata layers on gender, biological age and especially genetics or letting the CNN determine them by itself. We suggest disregarding the established cephalometric points, lines, angles and plains as these were defined in regards to lateral X-ray, emphasising good contrast of the bone structures with high reproducibility of the point and not necessarily with focus on particular structures most affected by growth. We suggest letting3D CNN establish its observations and focus areas.

We also suggest allowing 3D CNN analysis of genetic predisposition in a smart way: by analysis of possibly CBCT of the biological parents or preferably non-invasive face-scan providing at least facial shell data.

### 2.3. The Data Management

The processing of data in deep learning is crucial for the sufficient result of any neural network. Currently, most of the implementations depend on the dominant model-centric approach to AI, which means that developers spend most of their time improving neural networks.

**Figure 8:**
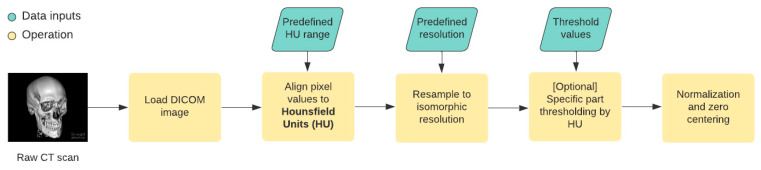
For medical images, there are various preprocessing steps that are recommended.

For medical images, various preprocessing steps are recommended. In most cases, the initial steps are following (Figure 8):

1. Loading DICOM files—the proper way of loading the DICOM file ensures that we will not lose the exact quality
2. Pixel values to Hounsfield Units alignment—the Hounsfield Unit (HU) measures radiodensity for each body tissue. The Hounsfield scale that determines the values for various tissues usually ranges from −1000 HU to +3000 HU, and therefore, this step ensures that the pixel values for each CT scan do not exceed these thresholds.
3. Resampling to isomorphic resolution—the distance between consecutive slices in each CT scan defines the slice thickness. This would mean a nontrivial challenge for the neural network. The thickness depends on the CT device setup, and therefore it is necessary to create equally spaced slices.
4. [Optional] Specific part segmentation—each tissue corresponds to a specific range in the Hounsfield units scale, and in some cases, we can segment out specific parts of the CT scan by thresholding the image.
5. Normalization and zero centering—these two steps ensure that the input data that are feed into the neural network are normalized into [0, 1] interval (normalization) and are zero centered (achieved by subtracting the mean value of the image pixel values).

Preprocessing the image dataset before feeding the CNN or other classifiers is important for all imaging modalities. Several preprocessing steps are recommended for the medical images before they are fed as input to the deep neural network model, such as (1) artefact removal, (2) normalization, (3) slice timing correction (STC), (4) image registration and (5) bias field correction. While all the steps (1) to (5), help in acquiring reliable results, STC and image registration are very important in the case of 3D medical images (especially nMR and CT images). Artefact removal and normalization are the most performed preprocessing steps across the modalities [146].

### 2.4. Dataset Specification

This study comprises approximately 500 iCAT CBCT scans of human heads. Each CBCT scan has the spatial resolution of 768 × 768 pixels and the default device pixel spacing is [0.3 × 0.3 × 0.3] millimeters.

The subjects are split by sex, with the ratio of 6:4 for female/male ranging from 8 to 72 years. The majority (90%) of the subjects are between 18 and 36 years.

These dataset parameters were used in suggested considerations for 3D CNN applications concepts stated in Section 2.2 Main tasks definitions.

### 2.5. Deep Learning Approach

#### 2.5.1. Age Estimation Using 3D Deep Neural Networks

In recent research [7,14] AI-based age estimation has proven to be a successful competitor to classical approaches from forensic medicine. The aim of this study is to create an automated system for age estimation from 3D cranial CT scans. There is an expectation that particular parts of the skull have a decisive impact on the final prediction, and therefore we propose a solution that includes two stages:

Age estimation from dense tissue layer—we use whole skull CT scan as an input into the 3D convolutional neural network, which would serve as a regression model that estimates the continuous values of age for each CT scan separately.

[Experimental] Visualization of network activations that represent regions of interest—neural network’s intermediate layers often serve as a an excellent explaining tool in order to find visual explanation heat maps [113] that highlight regions that affect neural network the most.

As for the specific neural network architecture, we derive the backbone part from the current state of the art research. We primarily consider the EfficientNet [147] and DenseNet [148] with their implementations adapted to 3D inputs.

Both architectures base includes convolutional layers that serve as feature extraction blocks to obtain specific indicators from input x represented as a loaded DICOM image. These extracted feature maps are then forwarded to a fully-connected layer that outputs the single age estimation value:

ŷ = F C(CL(x)) (1)

where CL is an intermediate block consisting of convolutional layers, FC is a fully-connected top part of the network that outputs a single floating-point value.

#### 2.5.2. Sex Classification Using 3D Deep Neural Networks

The determination of sex from human remains is a challenging task in various fields such as archeology, physical anthropology and forensics because there is no proven method that exactly leads to correct classification.

The use of AI in this field is highly desirable as manual determination is often very complex and time-consuming. The objectiveness of the deep learning approach can also eliminate human bias leading to reliable software products.

The sex classification is carried out similarly to the previous age estimation approach, but this task´s objective is to classify the final outputs from the neural network into 2 classes—female and male. For this purpose, we use the softmax activation function as a last operation to obtain class probabilities for both targets. The computation is following:

ŷ = α (FC(CL(x))) (2)

where CL and FC represent the convolutional and fully-connected blocks of the neural network. 

The experimental part would include the input x consisting of 2 separate inputs—one will be the segmented skull and the other will be the segmented soft tissue (skin) which is achieved by setting different thresholds for segmentation preprocessing step.

#### 2.5.3. Automatization of Cephalometric Analysis

The cephalometric analysis aims to set landmarks of CT(CBCT) scans which serve as an important factor in the alignment of a skull. These measurements can also be used as surgery planning parameters or pre-and post-surgery comparisons [149,150].

**Figure 9:**
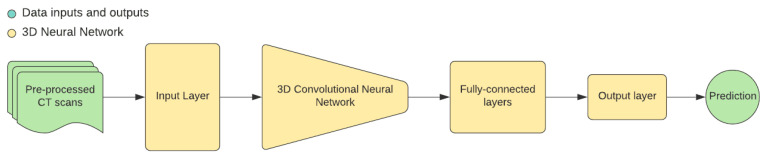
Pipeline from pre-processed CBCT scans to prediction on 3D CNN.

The idea behind this approach is to use 3D convolutional neural networks for fully automated cephalometric analysis. Networks aim to output probabilistic estimations for each cephalometric landmark and then create a projection of these estimations into a real skull CT scan (Figure 9).

Two approaches come into consideration:

1. Landmarks estimation in whole CT scan image—in this approach, the probability estimation for all landmarks is assigned for each pixel in the CT scan
2. Landmarks estimation for selected regions of interest—assuming that each landmark corresponds to a specific area we could add another preprocessing step—slice cut where each slice would be a template-based region fed into a neural network. We can determine the expected landmark detection for each slice independently, which should help in the final model performance

#### 2.5.4. Neural Networks Architectures and Clinical Data Pre-Processing

Recently, CNNs have been successfully applied in widespread medical image analysis and achieved significant benefits [9,59,115,141,151]. We investigated the design of a 3D CNN with backbones based on Resnet, MobileNet, and SqueezeNet models, which have proven to be the most efficient and widely used in various applications. One of the preferable architectures was based on 3D Resnet34 for the mandible segmentation in research of Pham et al. 2021 [113].

We have considered various approaches:

- Use whole 3D CT scan as an input into the neural network and output one value for age estimation as floating value and one for sex classification as a binary value.
- Segment out the mandible and use it as input into the neural network. Output is the same as in the previous task.
- (experimental) Use a whole 3D CT scan to input into the neural network and output multiple values representing specific skull features (as discussed at the meeting last week). Then use these values as an input into another machine learning model to estimate age and gender.

Suppose we take an example of mandible segmentation from DICOM. The first step is to have DICOM files loaded and then, added any missing metadata; particularly, the slice thickness, that is, the pixel size in the Z direction, which was obtained from the DICOM file. The unit of measurement in CBCT scans is the Hounsfield Unit (HU), which is a measure of radiodensity. Thus, HU shall be converted to pixel values. Subsequently, it shall be resampled to an isomorphic resolution to remove the scanner resolution. The slice thickness refers to the distance between consecutive slices (when viewing a 3D image as a collection of 2D slices) and varies between scans.

The final preprocessing step is bone segmentation and pixel normalization. Mandible bone extraction is complex because the surrounding bone has to be removed. An image binary thresholding and morphological opening operation for each slice shall be applied.

The morphological opening operation is an essential technique in image processing, achieved by erosion and the dilation of an image. This technique helps to remove small objects while retaining more significant parts from an image. To obtain the mandible bone part, the largest areas after morphological opening shall be kept. Finally, all the slices shall be stacked together to obtain the mandible voxels.

### 2.6. Evaluation

All approaches are evaluated in a classical machine learning manner—the dataset is split into three parts train, validation and test split. The test split mainly serves as a benchmarking set in order to compare our results with other approaches.

#### 2.6.1. Regression Models

When dealing with regression models in the deep learning field, we usually take into consideration two main regression metrics—Mean Absolute Error (MAE) and Mean Squared Error (MSE). Both metrics calculate the error between predicted y and ground truth labels denoted as y.

MAE is defined as the mean of the sum of absolute differences between y and ŷ for each pixel:

(3) $$\mathrm{MAE}=\frac{1}{n}\sum \left(y-ŷ\;\right)$$

while MSE is defined as mean of the squares of the errors, where error is defined as difference between y and ŷ:

(4) $$\mathrm{MSE}=\frac{1}{n}\sum {\left(y-ŷ\;\right)}^{2}$$

the regression tasks are primarily related to Task 1—age estimation and Task 3—automated cephalometric analysis.

#### 2.6.2. Classification Models

In order to evaluate the classification task, which in our case is represented by Task 2—sex classification, we need to consider the current distribution of male and female samples in our dataset. As the distribution is approximately 6:4 (almost equal), we can calculate the overall accuracy and corresponding confusion matrix (CM). In the later phase, we can also test other metrics such as precision, recall or F1 score.

**Figure 10:**
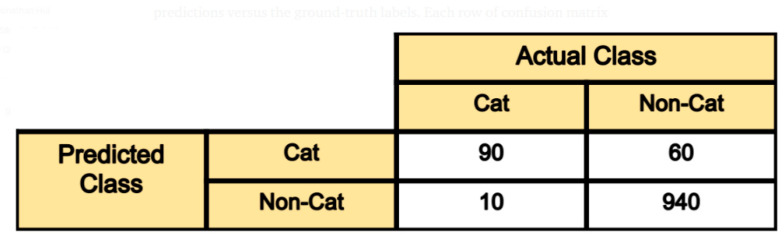
Confusion matrix for 2 classes image classification—Cat and Non-Cat. Each row corresponds to the predicted class from neural network output. In case of a class Cat 90 samples were correctly classified as Cat and 60 samples were incorrectly classified as Non-Cat.

The calculation of accuracy is defined just as the number of correct predictions divided by the total number of predictions. More interesting for use would be the CM. It is a tabular visualization of a model prediction for each class separately (Figure 10).

## 3. Resulting Summary of Proposed Approach for Utilization of 3D CNN in Investigated Aspects of Forensic Medicine

This chapter is presenting summary outcome from the detailed research in previous sections of this paper. Investigation of 3D CNN modalities, their features, advantages and disadvantages and also clinical requirements for implementation in the field of forensic medicine has led to these proposed designs (guide) of future forensic research based on 3D CNN analyses.

Table 2 presents condensed summary of recommended approach for 3D CNN implementations in various forensic topics. Expected input data is the minimal dataset of 500 full-head CBCT scans, described in more detail in previous sections.

## 4. Discussion

The authors of this paper have no doubts that 3D CNN, as another evolutionary step in advanced AI, will be with practical implementation a watershed moment in forensic medicine fields dealing with morphological aspects.

With considered data input as CT or CBCT (DICOM), the implementation of 3D CNN algorithms opens unique opportunities in areas of:

- Biological age determination
- Sex determination
- Automatized, precise and reliable:
  ○ 3D cephalometric analysis of soft and hard tissues
  ○ 3D face prediction from the skull (soft-tissues) and vice versa
  ○ Search for hidden damage in post-mortem high-resolution CT images
  ○ Asymmetry and disproportionality evaluation
- Predictions of:
  ○ Hard-tissue and soft tissue growth
  ○ Aging in general
  ○ Ideal face proportions respecting golden ratio proportions
- 3D reconstructions of:
  ○ Missing parts of the skull or face
  ○ 3D dental fingerprints for identification with 2D dental records

First clinical applications of 3D CNN have shown [91,113,115,126,150] that the algorithms can be successfully used in CT analysis and identifications of specific diseases such as Alzheimer or COVID19 as these have a specific representation on the X-ray. With a high probability bordering on certainty, the future development of advanced 3D CNN will result in sophisticated automatized algorithms processing 3D diagnostic data similarly to the trained human eye of the forensic expert. These algorithms will automatically process 3D diagnostic data such as CT or NMR, searching for patterns they were trained to see. They will recognize unseen details of hidden damage or representations of rare diseases when trained to do so. In the next level, they will approximate the finding to become an ultimate autopsy tool for even unknown diseases [36,113,126,152].

The limitation of this paper is that practical examination of the proposed directions for 3D CNN implementations will require some time. Currently, there are many different 3D CNN in development, and actually, this is where most of the research activity is carried out [151,153,154,155].

Another limitation of this study is the high level of dynamics of research and development in this field of advanced AI implementations. The velocity in training the 3D CNN is high, and it is possible that a better approach can be recognized in the process.

Interesting limitation of 3D CNN usage is the known fact [99] the any AI may become biased in the same way as a human forensic expert does and not only in the context of the criminal trial. This depends on the source data used for AI training [99] and is elaborated in more context in Section 1.2. On the other hand, in many forensic cases we need to achieve highest probabilities on the boundary with certainty. Here a respected and internationally recognized algorithm might become a useful tool for achieving an unprecedented levels of probability superior to human evaluation. However, this development is a possibility, not certainty.

The final limitation of implementing the suggested designs for 3D CNN implementation for forensic researchers is the physical and legal availability of big data necessary for 3D CNN training. This can be solved with multicentric cooperation.

There already exist many CNN processing DICOM data and are available for use [11,12,14]. Researchers this year have already achieved significant milestones in multiclass CBCT image segmentation for orthodontics with Deep Learning. They trained and validated a mixed-scale dense convolutional neural network for multiclass segmentation of the jaw, the teeth, and the background in CBCT scans [153]. This study showed that multiclass segmentation of jaw and teeth was accurate, and its performance was comparable to binary segmentation. This is important because this strongly reduces the time required to segment multiple anatomic structures in CBCT scans.

In our efforts, we have faced the issue of CBCT scan distortion caused by metal artefacts (mostly by amalgam dental fillings). Fortunately, a novel coarse-to-fine segmentation framework was recently published based on 3D CNN and recurrent SegUnet for mandible segmentation in CBCT scans. Moreover, the experiments indicate that the proposed algorithm can provide more accurate and robust segmentation results for different imaging techniques compared to the state-of-the-art models with respect to these three datasets [156].

As there already exists a fully automated method for 3D individual tooth identification and segmentation from dental CBCT [154], these algorithms can be combined.

The most complex area covered by this paper is a 3D prediction of growth and in a wider perspective of ageing. It is known that this process is laden with various variables including hormonal (sex) [142,143,157,158,159] and functional aspects (bad habits) [160,161,162], as well as genetics [163,164,165,166].

The only published study focused on predicting the facial growth direction with the implementation of Machine Learning methods is from 2021 Kazmierczak et al. 2021 [90,145]. The outcomes of this paper are limited in regards to facial growth prediction. The authors of this original paper did feature selection and pointed the attribute that plays a central role in facial growth. Then they performed data augmentation (DA) methods.

The principal weakness of this study is not the method but probably the input. The authors used only 2D lateral X-rays of various qualities and sizes. In addition, the evaluation was performed only in one 2D projection. The researchers focused on the angle between the Sella—Nasion line and the Mandibular plane formed by connecting the point gonion to gnathion at the inferior border of the mandible. They engaged an orthodontic expert to identify approximately 20 characteristic anatomic landmarks on LC to assess a subject. These were assessed manually on the lateral cephalogram. Some of the landmarks define angles which, from the clinical perspective, have special significance. As far as facial growth direction is concerned, there are no standardized measurements available in the literature to evaluate. The focus of supervised ML with a concentration on established cephalometric parameters might be wrong. It is the fact that they were originally chosen as well distinguished points on lateral X-ray with a priority of high reproducibility. So as considered by Hirschfeld and Moyers more than 50 years ago, we might be looking in the wrong places. Prediction of the change of SN/MP also oversimplifies the problem. The questions from the past remain, and facial growth prediction remains a complex mystery. The application of 3D CNN on this most complex task is described in more technical details and discussed later in the context of addressing other variables such as biological age, sex and genetics.

## 5. Conclusions

In conclusion, we can assume that the 3D CNN, as an advanced AI feature, will shift the paradigm in all areas researched in this paper. Forensic experts are now guided to step into the era of artificial intelligence as a helpful tool for research and possibly even future routine forensic analyses. Proposed methods and metrics for 3D CNN application on particular forensic topics (Biological age determination, Sex determination, 3D cephalometric analysis and Face prediction from skull), summarized in resulting Table 2, can be used as the initial guide. Forensic 3D reconstructions using artificial intelligence will be new, exciting and practically usable methods.

The implementation of advanced AI still requires interdisciplinary cooperation, albeit, with understanding, it can be used to crack unsolved mysteries. It definitely is not a trend that can be ignored.

## Figures and Tables

**Table 1:** Full-head CBCT scans were mate with i-CAT™ FLX V17 with these settings.

Parameter	Setting
Sensor Type	Amorphous Silicon Flat Panel Sensor with Csl Scintillator
Grayscale Resolution	16-bit
Voxel Size	0.3 mm,
Collimation	Electronically controlled fully adjustable collimation
Scan Time	17.8 s
Exposure Type	Pulsed
Field-of-View	23 cm × 17 cm
Reconstruction Shape	Cylinder
Reconstruction Time	Less than 30 s
Output	DICOM
Patient Position	Seated

**Table 2:** Guide of recommended designs for 3D CNN implementations in various forensic topics.

Area of Forensic Research	Proposed Method	Metrics
Biological age determination	Regression model by 3D deep CNN	MAE, MSE
Sex determination	Deep 3D CNN—conv.layers and outputs class probabilities for both targets	CM such as precision, recall and F1 score
3D cephalometric analysis	Object detection model on 3D CNN that auto.estimates cephalom.measurements	MAE, MSE
Face prediction from skull	model on Generative Adversarial Network that synthesize soft/hard tissues	slice-wise Frechet Inception Distance
Facial growth prediction	Based on methods stated above ^1^	another ^1^

^1^ Method and metrics are not proposed from the current state of knowledge for Facial growth prediction and need further consideration upon clinical experience from 3D CNN applications.

## Data Availability

We fully adhere to Data Availability Statements in section “MDPI Research Data Policies” at https://www.mdpi.com/ethics (accessed on 1 November 2021).

## Abbreviations

3D CNN	Three-Dimensional Convolutional Neural Network
AI	Artificial Intelligence
CBCT	Cone-Beam Computer Tomography
CM	Confusion Matrix
DICOM	Communications in Medicine Format
DA	Data Augmentation
FOV	Field of View
HU	Hounsfield Unit
ML	Machine Learning
MAE	Mean Absolute Error
MSE	Mean Squared Error
nMR	Nuclear Magnetic Resonance
STC	Slice Timing Correction
